# Using targeting to recruit men and women of color into a behavioral weight loss trial

**DOI:** 10.1186/s13063-020-04500-1

**Published:** 2020-06-16

**Authors:** Melissa M. Crane, Elisabeth M. Seburg, Rona L. Levy, Robert W. Jeffery, Nancy E. Sherwood

**Affiliations:** 1 Department of Preventive Medicine, Rush University Medical Center, 1700 W. Van Buren St., Suite 470, Chicago, IL 60612 USA; 2 HealthPartners Institute, 8170 33rd Ave South, Minneapolis, MN 55440-1524 USA; 3 School of Social Work, University of Washington, 4101 15th Avenue NE, Seattle, WA 98105-6250 USA; 4 Division of Epidemiology and Community Health, University of Minnesota, 300 West Bank Office Building, 1300 S. 2nd St., Minneapolis, MN 55454 USA

**Keywords:** Targeted recruitment, Weight loss, Men, People of color

## Abstract

**Background:**

The majority of participants in weight loss trials are non-Hispanic White women, while men and women of color are underrepresented. This study presents data obtained from non-targeted and targeted recruitment approaches in a trial of behavioral weight loss programs to (1) describe the yields from each approach and (2) compare the demographics, weight control histories, and study involvement of samples recruited by each approach.

**Methods:**

Data for this observational study include source of recruitment, demographic information, weight loss experiences (e.g., lifetime weight loss, current weight loss behaviors), and completion of the 6-month assessment visit.

**Results:**

Men comprised 14.2% of participants who responded to non-targeted recruitment efforts, while targeted efforts yielded 50.4% men. Similarly, people of color comprised 12.8% of those who responded to non-targeted approaches, whereas targeted recruitment methods yielded 47.2% people of color. Men recruited through targeted methods were younger (*p* = 0.01) than men recruited through non-targeted means but were otherwise similar. Women of color recruited through targeted methods reported use of fewer weight loss strategies relative to women of color recruited through non-targeted means (*p* = 0.006) but were otherwise similar. There were no differences by recruitment method on retention to the study.

**Conclusions:**

Using targeted recruitment methods increased the ethnic and gender diversity of the recruited sample without reducing study retention. This targeting also increased the enrollment of women with less weight loss experience who may not have otherwise sought out a weight loss program. Developing and implementing a targeted recruitment plan should be considered early in the clinical trial development process.

**Trial registration:**

Clinicaltrials.gov, NCT02368002. Registered on 20 February 2015.

## Background

Recruiting and retaining diverse samples are vital to conducting externally valid studies [1]. However, most areas of research have groups that are underrepresented. Within the area of behavioral weight control, samples are predominately female [2–5] and non-Hispanic White [5, 6]. These participants do not mirror the population with obesity in the USA because the prevalence of obesity is high in all racial and ethnic groups, except Asian Americans, and is highest among African American women [7]. Further, the prevalence of obesity is similar in men (37.9%; 95% CI 33.1–42.8) and women (41.1%; 95% CI 37.8–44.5) [8]. In order for study participants to better represent the populations that need weight control programs, calls for greater recruitment of men and people of color into weight loss programs have been made [2, 3, 6].

To reduce the homogeneity of participants in behavioral weight loss intervention trials, researchers have used targeted messaging to increase the personal relevance of the recruitment messages. Theory suggests that targeting the content of a message to increase its personal relevance will increase the likelihood that the person will attend to and process the message [9]. For example, a person may provide greater attention to an advertisement if it mentions their gender or ethnic identity. Similarly, if an advertisement mentions a behavior they are already engaged in, a person is more likely to attend to the message. Targeted health promotion messages are more effective than non-targeted messages across multiple health behaviors [9–11]. Recommendations for targeting in recruitment efforts have included targeting the messages to the subgroup of interest, such as emphasizing the importance of the health issue for an ethnic group, or targeting where the message is placed, such as mailing letters directly to individuals from the targeted subgroup [6, 12, 13]. In randomized comparisons of printed recruitment efforts, targeting recruitment materials has generally led to an increase in recruitment among women of color [14–16] and a slight increase in recruitment of men [17] into behavioral weight control trials.

Despite reports focusing on ethnic and gender composition of the study samples recruited, there is scant information about whether those recruited through these targeted channels differ on any other characteristics, such as prior experience with behaviors relevant to the study or other demographic characteristics. This is an important consideration because there is some evidence that the content of a recruitment message may influence who responds. For example, one study focused on mental health and well-being for men found that recruitment messages emphasizing mental strength had the greatest reach and were most effective at reaching young men. Meanwhile, the advertisements focused on “mental health” yielded a smaller sample, but a sample with greater depressive symptoms, one target of the intervention [18]. Studies from two weight loss trials [19, 20] have previously reported that participants have significantly greater weight loss experience than non-participants [21, 22], but no studies have investigated whether weight loss experience varies by source of recruitment. Theory suggests that individuals already engaged or thinking about weight loss may be more responsive to non-targeted advertisements, but this has not been demonstrated. Engaging individuals with less weight loss experience may be important given the health and psychological benefits of participating in behavioral weight loss programs [23, 24].

With the ultimate goal of increasing the heterogeneity of participants, there is a preliminary need to better characterize samples recruited into clinical trials via different recruitment approaches. The current study aims to address the need for characterization by describing different recruitment efforts for a behavioral weight loss trial, and the resulting samples. Recruitment efforts included both non-targeted recruitment approaches (i.e., not designed to reach any specific subgroups) and approaches that used targeted content and targeted placement of advertisements focused on reaching men and people of color, referred to throughout the manuscript as “targeted” recruitment approaches. We describe our recruitment approaches, the yields of these efforts, and then characterize the resulting samples at both the initial contact and the sample that was randomized into the trial. We hypothesized that participants who first heard about the study through non-targeted means would more likely be women, non-Hispanic White. We hypothesized that individuals recruited through non-targeted means would be more likely to be already engaged in weight control behaviors. We also explored whether the samples varied on other demographic characteristics or retention into the study.

## Methods

### Study overview

The data for this study come from the BestFIT (Finding Individualized Treatments) study (NCT02368002). This study is a sequential multiple assignment randomized trial testing treatment options for suboptimal responders to behavioral treatment for weight loss [25]. Briefly, participants were recruited to take part in 6 months (20 weeks) of behavioral weight loss intervention delivered via one-on-one coaching sessions with health education specialists such as dieticians and public health educators. Major eligibility criteria for the study included age 21–70 years, BMI 30.0 to 45 kg/m^2^, and willingness to engage with the study for 18 months [25]. Major exclusion criteria included inability to safely participate in physical activity, pregnancy or breastfeeding or planning a pregnancy within the next 18 months, involvement in another diet intervention or weight loss program, dietary restrictions (e.g., gluten-free), insulin-dependent diabetes, and the presence of a significant psychiatric disorder (e.g., schizophrenia) that could interfere with trial participation [25]. Participants completed study assessment visits prior to treatment, post-treatment (6 months), and 1 year post-treatment (18 months). Assessment visits were conducted in-person at the research site and included measurement of height and weight, completion of online surveys, and other measurement tasks. A full description of the assessment is available elsewhere [25]. Recruitment was conducted between May 2015 and August 2017. Consent was provided verbally for the telephone screening, and written consent was collected during the baseline assessment visit.

### Recruitment

Two general types of recruitment strategies were utilized in this study: non-targeted and targeted. *Non-targeted recruitment* efforts did not focus on any specific demographic groups and included posting advertisements on the employee-facing website for a large health insurance and healthcare system, posting recruitment information on the social media page of the health system, placing notices in health system member newsletters, and word-of-mouth. The recruitment messaging used in these advertisements included language such as “Tired of the one-size-fits-all approach to weight loss? The HealthPartners Institute is currently looking for people for the BestFIT study....” (see Supplemental File for full text of advertisements). Participants could also self-refer to the study from online sources such as the study’s registration on ClinicalTrials.gov and online search results that led participants to the study website. Few participants reported sources of recruitment that do not fall into any formal category, and they were classified as “other” sources (e.g., press release, meal vender used in the study). There were no additional costs for the non-targeted recruitment strategies.

*Targeted recruitment* efforts were used to improve recruitment of men and people of color into the study, and included targeting the recruitment message content and the message placement to reach these audiences. To reach men, radio advertisements were developed that were directed towards men with the message: “Guys: if you let out your belt instead of tightening it; if your game is getting slower, it is time to boost your fitness and your health by losing a few pounds for free … ..” (full script provided in the Supplemental File). These advertisements aired on a local sports radio station with a high percentage of male listeners. The total cost to produce and broadcast these advertisements was $7000. Additional advertisements were developed that were generic in language and aired on stations with a high percentage of listeners of color (full script provided in the Supplemental File). The total cost for these ads was $6300. Recruitment letters (*N* = 3000) were targeted in both messages and placement and were sent to patients of the healthcare system that were either men of any ethnic background and women of color with overweight or obesity. These letters included language that the letters were being sent because they had seen a HealthPartners physician in the past year and because the study was seeking “men and women from diverse backgrounds to participate” in the study. The cost to prepare and deliver these letters was $3105 (which includes programming staff time, staff time for preparing the mailings, stationery, and postage). In-clinic advertisements were included on notice screens in waiting rooms in health clinics that served primarily patients of color. This approach utilized targeted placement and generic wording (“Weight loss: one size does not fit all: HealthPartners Institute is conducting a FREE weight loss study to learn more about personalized programs. We’re looking for participants ages 21 to 70 – see if you’re eligible!”). Finally, notices about the study were included in church bulletins of local churches with primarily congregants of color, though this method did not yield any participants. There were no additional costs associated with the in-clinic advertisements and church bulletins.

For all modes of recruitment, potential participants called the study staff and completed a brief telephone screening. Participants attended a group orientation session where the study was described. Participants returned for a baseline study visit where written consent was obtained and were then randomized into the trial.

### Measures

*Recruitment source* was assessed during the telephone screen when participants were asked “How did you hear about the study?” Full responses were recorded and later classified as either non-targeted (e.g., employee-facing advertising, word-of-mouth, other) or targeted (radio, letters, or in-clinic ads). Very few participants (*n* = 12, 0.97%) did not report how they heard about the study and were classified being recruited through a non-targeted source.

*Age*, *gender*, *and self-report weight and weight* were collected during the telephone screen (*N* = 1243). Participant ethnicity was collected only during the final third of recruitment (*n* = 484). Racial and ethnic groups were divided into non-Hispanic White and people of color (including Hispanic of any racial classification, Black/African American, Asian, Native American, multiple races, and other).

*Objective weight and height* were assessed among randomized participants (*N* = 468) during the baseline assessment visit with the participant wearing light street clothing with shoes removed. Ethnic identity, highest level of education completed, and household income were collected via self-report. Due to distribution of responses, education level and household income were recoded into completing less than college degree or greater and income less than $75,000 or greater.

*Weight loss behaviors* were self-reported at baseline including lifetime weight loss, weight loss strategy use, and frequency of self-weighing. The weight loss history questionnaire assessed the number of times (0 times, 1–2 times, 3–4 times, 5–6 times, 7+ times) that the participant had intentionally lost (1) 5–9 pounds, (2) 10–19 pounds, (3) 20–49 pounds, (4) 50–79 pounds, (5) 80–99 pounds, and (6) 100+ pounds. To create a lifetime total weight loss, the mean values for each weight loss range and each response option were multiplied (e.g., 1.5 times × 34.5 pounds = 51.75 pounds) and then summed [26]. Current use of weight loss strategies was assessed through a nine-item measure. This inventory asked participants how frequently (7-point response: “Never” to “Very Often”) they used the following weight loss strategies: ate fresh, low-calorie entrees; ate frozen, low-calorie entrees; used liquid meal replacement products; used powder meal replacement products; used meal replacement bars; planned meals; planned exercise; record calories eaten; and record exercise. A total strategy use was calculated by summing the number of strategies participants reported using “often” or “very often” (possible range 0 to 9). Finally, self-weighing frequency was assessed using a single item question with seven response options from “never” to “more than once per day.” This item was dichotomized as less than once a week or once per week or more [27].

*Study involvement* was assessed using three variables appropriate for the stage of the study. Study status after the telephone screen was categorized as eligible, ineligible, or no longer interested. Between the telephone screen and randomization, participants could choose to stop involvement in the study prior to randomization (e.g., by not completing the baseline assessment). Thus, continued involvement in the recruitment process was measured via randomization into the study (yes/no). Finally, as a concise way to investigate whether recruitment source influenced longer-term involvement in the study, completion of the 6-month assessment was used as a proxy. Here, completion of the assessment was defined by at minimum providing a weight during the 6-month assessment window.

### Analysis

To investigate the impact of targeted versus non-targeted recruitment efforts, we began by describing the number of telephone screens completed by recruitment source and the gender and ethnic composition of the yields (Supplemental Table). Using the sample who completed the telephone screen, we compared those recruited via the targeted and non-targeted methods on gender, ethnicity, age, BMI, eligibility study after the telephone screen, and randomization into the study. Next, we compared the randomized participants by their recruitment source comparing those recruited via non-targeted or targeted methods. Because the overall sample contains a high proportion of non-Hispanic White women, we compared men recruited through targeted versus non-targeted means and women of color recruited through the two approaches. Comparisons of the two recruitment approaches were made using chi-square and independent *t* tests, as appropriate. Self-reported BMI from the telephone screening participants and lifetime weight loss among the randomized participants were positively skewed and were tested using the Wilcoxon-Mann-Whitney *U* tests. Analyses were conducted with and without one participant with an extreme value on total weight loss. Results were similar and the data presented exclude this participant.

## Results

**Fig. 1:**
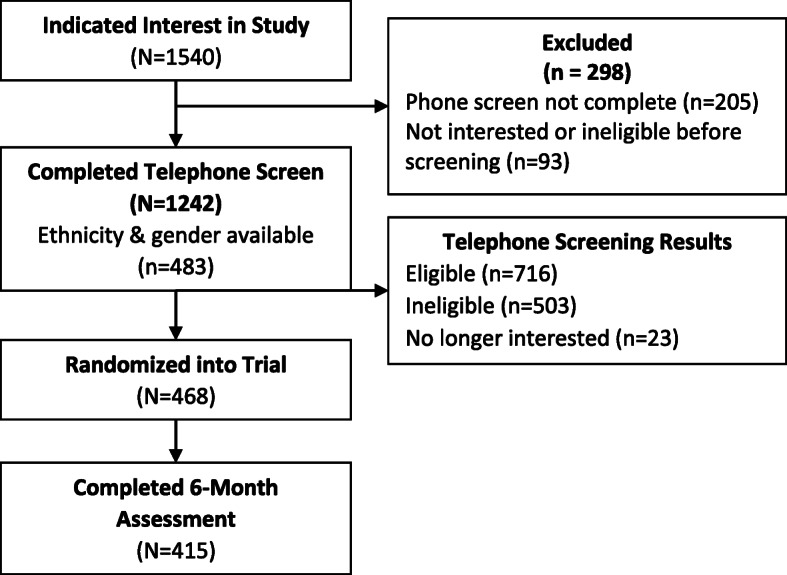
Participant flow through the 6-month assessment

As shown in Fig. 1, 1540 individuals contacted the study during the recruitment period. Of those, 1242 completed the telephone screen and answered the recruitment source question. Reasons for not completing this call included the following: (1) unable to be contacted (*n* = 205), (2) expressed disinterest in the study prior to the screening questions (*n* = 34), or (3) were deemed ineligible prior to screening (e.g., revealed unable to attend meetings; *n* = 59). The Supplemental Table shows the number of individuals who heard about the study through all recruitment sources. The yield is presented by gender for all potential participants screened and separately for the subset who had both ethnicity and gender available during the telephone screening (*n* = 483) and those randomized into the study. Descriptively, the non-targeted recruitment efforts yielded greater numbers of women and non-Hispanic White participants while the targeted recruitment efforts (radio advertisements, letters, in-clinic advertisements) yielded a greater proportion of men and people of color, as planned.

Among those who completed the telephone screen, we then investigated whether there were differences between participants who were recruited via targeted methods versus non-targeted methods on gender, ethnicity (where available), age, BMI, eligibility for the study after the telephone screen, and randomization. As shown in Table 1, participants recruited via targeted recruitment efforts were more likely to be men (*χ*^2^ = 198.0, *p* < 0.001) and people of color (*χ*^2^ = 145.1, *p* < 0.001). They were also younger (*t* = 5.31, *p* < 0.001). There were no differences on BMI (*z* = − 0.47 *p* = 0.64) or randomization rates (*χ*^2^ = 1.76, *p* = 0.18).

**Table 1:** Comparison of telephone screening respondents (*N* = 1242) by recruitment source

	*N*	Non-targeted recruitment	Targeted recruitment	*p* value
Gender, *n* (%)	1238			< 0.001
Woman		773 (87.9)	183 (51.0)	
Man		106 (12.1)	176 (49.0)	
Ethnicity, *n* (%)	483			< 0.001
Non-Hispanic White		243 (80.7)	46 (25.3)	
Person of color		58 (19.3)	136 (74.7)	
Age, year, M ± SD	1238	48.2 ± 11.4	44.4 ± 11.2	< 0.001
Self-reported BMI, kg/m^2^, mdn (IQR)	1229	34.5 (30.9; 39.2)	34.5 (31.2; 38.0)	0.64
Eligibility after telephone screen, *n* (%)	1242			0.10
Eligible		496 (56.2)	220 (61.3)	
Ineligible		373 (42.3)	130 (36.2)	
No longer interested		14 (1.6)	9 (2.5)	
Randomization status, *n* (%)	1242			0.18
Randomized		343 (38.8)	125 (34.8)	
Not randomized		540 (61.2)	234 (65.2)	

Values are observed means and standard deviations, median and interquartile range, or counts and percentages

### Randomized participants

Four hundred sixty-eight participants were randomized into the study. The ethnic and gender composition of the final study sample included 58.6% non-Hispanic White women, 19.7% non-Hispanic White men, 17.7% women of color, and 4.1% men of color. Compared to participants recruited through non-targeted means, participants recruited through targeted methods were more likely to be men (Table 2; *χ*^2^ = 63.15, *p* < 0.001) and people of color (*χ*^2^ = 64.58, *p* < 0.001). They were also younger (*t* = 2.85, *p* = 0.005), were less likely to be employed full-time (*χ*^2^ = 5.42, *p* = 0.02), and reported less lifetime weight loss (*z* = − 3.29, *p* = 0.001) and lower current use of weight loss strategies (*t* = 3.88, *p* < 0.001). There were no differences on study retention at 6 months (*χ*^2^ = 0.37, *p* = 0.54). Among men randomized into the study (Table 3), those who were recruited via targeted methods were younger (*t* = 2.53, *p* = 0.01) than those recruited via non-targeted methods; no other differences were significant. Among women of color (Table 4), those recruited by targeted methods reported lower use of weight loss strategies (*t* = 2.81, *p* = 0.006); no other differences were significant.

**Table 2:** Comparisons of demographic characteristics, weight loss experience, and completion of the 6-month assessment of all randomized participants by recruitment source

	Non-targeted recruitment, *n* = 343	Targeted recruitment, *n* = 125	*p* value
Demographics			
Women, *n* (%)	294 (85.7)	63 (50.4)	**< 0.001**
Non-Hispanic White, *n* (%)	300 (87.5)	66 (52.8)	**< 0.001**
Age, M ± SD	49.8 ± 10.3	46.7 (10.3)	**0.005**
Objective BMI, M ± SD	36.0 ± 3.8	35.9 ± 4.0	0.82
Income ≥ $75,000, *n* (%)^a^	221 (64.8)	68 (54.8)	0.05
≥ College degree, *n* (%)	205 (59.8)	76 (60.8)	0.84
Employed full-time, *n* (%)^b^	294 (85.7)	95 (76.6)	**0.02**
Married/partnered, *n* (%)^b^	238 (69.6)	82 (65.6)	0.41
Weight loss experience			
Lifetime total weight loss, lbs., mdn (IQR)^c^	126.0 (74.3; 224.0)	98.0 (38.5; 180.8)	**0.001**
Total weight loss strategies, M ± SD	3.3 ± 1.7	2.6 ± 1.8	**< 0.001**
Self-weighing frequency ≥ weekly, *n* (%)^b^	171 (50.0)	55 (44.0)	0.25
Complete 6-month assessment, *n* (%)	306 (89.2)	109 (87.2)	0.54

Values are observed means and standard deviations, counts and percentage, or median and interquartile range (total weight loss only)

^a^*n* = 465

^b^*n* = 467

^c^*n* = 466

**Table 3:** Comparisons of demographic characteristics, weight loss experience, and completion of the 6-month assessment of randomized participants by recruitment source: men only (*N* = 111)

	Non-targeted recruitment, *n* = 49	Targeted recruitment, *n* = 62	*p* value
Demographics			
Non-Hispanic White, *n* (%)	41 (83.7)	51 (82.3)	0.84
Age, M ± SD	52.3 ± 9.0	47.5 ± 10.6	**0.01**
Objective BMI, M ± SD	35.7 ± 3.9	35.9 ± 3.9	0.72
Income ≥ $75,000, *n* (%)	42 (85.7)	44 (71.0)	0.06
≥ College degree, *n* (%)	40 (81.6)	43 (69.4)	0.14
Employed full-time, *n* (%)^a^	45 (91.8)	51 (83.6)	0.20
Married/partnered, *n* (%)	40 (81.6)	47 (75.8)	0.46
Weight loss experience			
Lifetime total weight loss, lbs., mdn (IQR)	126.0 (51.8; 213.0)	103.3 (51.8; 84.0)	0.56
Total weight loss strategies, M ± SD	3.0 ± 1.6	2.4 ± 1.8	0.08
Self-weighing frequency ≥ weekly, *n* (%)	25 (51.0)	27 (43.6)	0.43
Complete 6-month assessment, *n* (%)	47 (95.9)	56 (90.3)	0.26

Values are observed means and standard deviations, counts and percentage, or median and interquartile range (total weight loss only)

^a^*n* = 110

**Table 4:** Comparisons of demographic characteristics, weight loss experience, and completion of the 6-month assessment of randomized participants by recruitment source: women of color only (*N* = 83)

	Non-targeted recruitment, *n* = 35	Targeted recruitment, *n* = 48	*p* value
Demographics			
Age, M ± SD	45.5 ± 10.5	46.2 ± 10.4	0.78
Objective BMI, M ± SD	36.6 ± 4.0	35.8 ± 4.0	0.37
Income ≥ $75,000, *n* (%)^a^	11 (31.4)	17 (36.2)	0.65
≥ College degree, *n* (%)	18 (51.4)	25 (52.1)	0.95
Employed full-time, *n* (%)	28 (80.0)	34 (70.8)	0.34
Married/partnered, *n* (%)	18 (51.4)	26 (54.2)	0.81
Weight loss experience			
Lifetime total weight loss, lbs., mdn (IQR)^a^	74.3 (32.3; 184.0)	51.8 (10.5; 147.5)	0.50
Total weight loss strategies, M ± SD	3.8 ± 1.8	2.7 ± 1.8	**0.006**
Self-weighing frequency ≥ weekly, *n* (%)	14 (40.0)	22 (45.8)	0.60
Complete 6-month assessment, *n* (%)	28 (80.0)	41 (85.4)	0.52

Values are observed means and standard deviations, counts and percentage, or median and interquartile range (total weight loss only)

^a^*n* = 8

## Discussion

This analysis confirmed that using targeted recruitment efforts increased the representation of targeted populations in a clinical trial. As compared to the sample recruited through non-targeted methods, the randomized participants recruited through the targeted approaches were far more diverse in terms of gender (49.2% men versus 12.1%) and ethnicity (12.5% people of color versus 47.2% people of color). This supports previous studies that indicated the utility of using printed targeted materials to recruit for weight loss programs [14, 15, 17].

A unique contribution of this manuscript is the analysis of characteristics of the sample by type of recruitment. Most importantly, in all analyses, the samples recruited by both targeted and non-targeted means were equally likely to remain in the study through the 6-month assessment. This suggests that expanding the recruitment approaches will not necessarily adversely affect study retention by increasing the heterogeneity of the sample. Additionally, the participants recruited through targeted recruitment methods were also younger, were less likely to be employed full-time, and had less weight loss experience. Because it is important to reach those with less weight loss experience [24] and because younger participants and participants with lower socioeconomic status are underrepresented in behavioral weight loss trials [28–30], these benefits further support the use of targeted recruitment efforts in future clinical trials beyond increasing the ethnic and gender composition of the sample.

The outcomes of this study and the experiences of recruiting these participants provide additional information for study designers interested in increasing representation in clinical trials. First, though the targeted methods used in this study were effective, they were much more costly than non-targeted recruitment. The overall cost of the targeted recruitment methods in this study was $16,404, and the average cost to recruit a randomized participant through the targeted channels was $133. By comparison, there were no additional costs (beyond staff time) to develop and disseminate the non-targeted recruitment methods. Few other manuscripts have published their recruitment costs per participant [31], and direct comparisons across studies are challenging due to differences in how costs are calculated (e.g., inclusion or exclusion of staff time). In this study, we used a patient database to identify letter recipients. This increased the cost of staff and programmer time but may be less costly than purchasing lists from external vendors. However, acknowledging that recruitment of underrepresented populations will likely require additional considerations for budget and time is vital to successful recruitment of diverse samples [13, 31, 32].

The second consideration for investigators hoping to enroll a more diverse sample is how targeting will be used. The recruitment messages used in this study relied primarily on targeted placement (in-clinic ads; selected radio stations) or surface-level targeting (including language mentioning the targeted groups in the letters). As suggested elsewhere, additional targeting of the messages to the population may enhance recruitment efforts [12, 13, 33].

Finally, this study began recruitment using non-targeted methods without formally tracking response rates of people of color. Tracking response rates to specific recruitment methods will help guide how to best use money for additional recruitment efforts and has been used in prior studies to change recruitment techniques to maximize effectiveness [34]. In addition to tracking recruitment, starting with targeted recruitment early in the recruitment process may maximize the recruitment effects. In this study, word-of-mouth was a common recruitment source. If targeted recruitment is started early in the study timeline, there would be added time for word-of-mouth recruitment to occur within the underrepresented populations. Although word-of-mouth recruitment may not be feasible or desirable in all studies due to clustering of cases, it may increase trust in the study and enhance participation in populations where mistrust of researchers has been well documented [35–37].

There were a number of limitations to this study. First, we were not able to randomize participants to receive either a targeted or a non-targeted message, thus limiting causal inferences which could be made. Second, recruitment took place at only one research site; therefore, we cannot comment on whether the results would be similar in other geographic areas. Third, the comparison of the relative costs of the two recruitment methods is limited, as non-targeted recruitment relied upon existing communication channels in a large workplace, allowing for message dissemination at no cost to the study. Despite these limitations, one particularly noteworthy strength of this study is that it compared recruitment efforts for a longitudinal, clinical trial rather than recruitment for a cross-sectional study with lower response burden.

## Conclusions

By using targeted recruitment efforts, including targeted radio advertisements and direct mail, this study was able to engage and retain men and people of color in a weight loss intervention trial. Building on these findings, future recruitment efforts should continue to use the effective strategies described here while also working with underrepresented groups to find additional channels of communication to enhance recruitment efforts. In addition, future research should use randomized comparisons to explore the effectiveness of best recruitment practices for underrepresented groups which may build on the findings provided in this study to test optimal recruitment strategies for randomized controlled trials.

## Supplementary information

**Additional file 1.**

## Data Availability

The datasets used and/or analyzed during the current study are available from the corresponding author on reasonable request.
