# Supplementary material for: Using targeting to recruit men and women of color into a behavioral weight loss trial
Source: Trials. 2020 Jun 16;21:537. doi: 10.1186/s13063-020-04500-1 (PMC7298816; doi:10.1186/s13063-020-04500-1)
Supplement: Supplementary file 1 — Additional file 1. [file 13063_2020_4500_MOESM1_ESM.pdf]

Supplemental File

Section 1. Text from recruitment materials

Supplemental Table. Yields by recruitment source during telephone screening and randomization.

Section 1. Text from recruitment materials

Generic Recruitment Text

Tired of the one-size-fits-all approach to weight loss? The HealthPartners Institute is currently looking for men and women for the BestFIT study. The goal of the study is to learn how to personalize weight loss programs. If you are interested and eligible, you would work one-on-one, in person, with a weight-loss coach at our Bloomington location. Depending on your progress, you may be randomly selected to receive pre-portioned meals or a different treatment therapy to help you lose more weight. This study is funded by the National Institutes of Health. To learn more about the BestFIT study, email us at [redacted], call us at [redacted]-[redacted], or go to [redacted].

Targeted Radio Ad- Men

Guys, if you let out your belt instead of tightening it or if your game is getting a little slower, it just might be time to boost your fitness and your health by losing a few pounds. For free. Join the HealthPartners' Institute Best Fit weight loss study! If you're between 21 and 70 and in generally good health, you may be eligible. In the BestFIT study, you'll meet, once a week, in Bloomington for personalized , one-on-one coaching. And, again, it's all free. To learn more, go to [redacted]. That's [redacted]

Targeted Radio Ad- People of Color

How you lose weight is up to you. You could spend money on an expensive program or lose weight for free. If you're between 21 and 70 and in generally good health, you might be eligible to join HealthPartners Institute's BestFIT weight loss study! Once a week, you'll meet, one-on-one in Bloomington, with a weight loss coach. This is no "one size fits all" approach, the BestFIT study provides personalized coaching to help you lose weight. And, again, it's all free. To learn more, go to [redacted]. That's [redacted].

Targeted Recruitment Letter Text

Dear <insert patient name>,

We are contacting you about an exciting research opportunity! The HealthPartners Institute is conducting the BestFIT (**F**inding **I**ndividualized **T**reatments) study to learn more about the best ways to help people lose weight. You are receiving this invitation because you had a visit to one of our HealthPartners clinics during the past year.

We are looking for men and women from diverse backgrounds to participate in this **free** 18-month weight loss study. All individuals who participate in the study will have 20 weekly, one-on-one meetings with a weight-loss coach at our Bloomington location near the Mall of America. Depending on your results, you may be chosen to try one of two different weight loss methods to help you lose more weight. You may be eligible to participate if you are:

- 21-70 years old
- Generally healthy
- Not allergic to any foods
- Not currently in a weight loss program or taking weight loss medications
- Not currently pregnant or planning a pregnancy in the next 18 months
- Between 20-80 pounds overweight for your height
- Willing to attend 20 in-person, weekly sessions at our Bloomington location, near the Mall of America
- Available to attend weekly sessions Monday - Friday between 9am and 3pm

Study participants will receive up to $85 in Target gift cards for helping us evaluate the effectiveness of the program.

If you are interested in participating in the BestFIT study, please call us at [redacted] or email us at [redacted], to learn more about the study and determine if you might be eligible to participate. You can also learn more about the study at our website: [redacted]. Taking part in this study is voluntary. Your decision will not affect the care you or your family receives at HealthPartners.

Thank you for your time and consideration.

Supplemental Table. Yields by recruitment source during telephone screening and randomization.

	Non-Targeted Recruitment							Targeted Recruitment		
	Word-of Mouth	Employee	Online[a]	Healthcare Provider	Ads to Plan Members	Other	Unknown	Strategic Radio	Strategic Direct Mail	Strategic Clinic Ads
All telephone screens (N = 1242)										
n	449	198	138	60	15	10	12	201	145	14
Women	391 (87.1)	185 (93.4%)	116 (84.7%)	52 (86.7%)	15 (100%)	10 (100.0%)	4 (40.0%)	59 (29.4%)	112 (77.8%)	12 (85.7%)
Men	58 (12.9)	13 (6.6)	21 (15.3)	8 (13.3)	0	0	6 (60.0%)	142 (70.7)	32 (22.2)	2 (14.3)
Ethnicity available at phone screen (N = 483)										
n	198	34	53	12	1	1	2	25	143	14
NHW Women	131 (66.2)	27 (79.4)	35 (66.0)	7 (58.3)	1 (100.0)	1 (100.0)	1 (50.0)	7 (28.0)	3 (2.1)	7 (50.0)
NHW Men	28 (14.1)	5 (14.7)	5 (9.4)	2 (16.7)	0	0	0	13 (52.0)	15 (10.5)	1 (7.1)
Women of Color	37 (18.7)	1 (2.9)	9 (17.0)	3 (25.0)	0	0	1 (50.0)	2 (8.0)	108 (75.5)	5 (35.7)
Men of Color	2 (1.0)	1 (2.9)	4 (7.6)	0 (0)	0	0	0	3 (12.0)	17 (11.9)	1 (7.1)
Randomized participants (N = 468)										
n	192	71	47	22	7	0	4	64	59	2
NHW Women	142 (74.0)	60 (84.5)	34 (72.3)	15 (68.2)	7 (100)	0	1 (25)	13 (20.3)	1 (1.7)	1 (50.0)
NHW Men	22 (11.5)	6 (8.5)	7 (14.9)	3 (13.6)	0	0	3 (75)	41 (64.1)	10 (17.0)	0
Women of Color	23 (12.0)	5 (7.0)	4 (8.5)	3 (13.6)	0	0	0	6 (9.4)	41 (69.5)	1 (50.0)
Men of Color	5 (2.6)	0	2 (4.3)	1 (4.6)	0	0	0	4 (6.3)	7 (11.9)	0

Note. Values are count and percentage within recruitment source. [a]Online recruitment included clinicaltrials.gov registry, Facebook posts by HealthPartners, and online search results.
